# The Impact of Climate Change on Road Traffic Crashes in Ghana

**DOI:** 10.21203/rs.3.rs-4654960/v1

**Published:** 2024-08-14

**Authors:** Ruth Akorli, Philip Antwi-Agyei, Patrick Davies, James Damsere-Derry, Frank Baffour-Ata, Emmanuel Nakua, Peter Donkor, Charles Mock

## Abstract

Despite the substantial injuries and fatalities from Road Traffic Crashes (RTCs), evidence of climate change's impact on RTCs in Ghana is lacking. This study assessed the impact of climate change on RTCs in Ghana by combining quantitative (Mann-Kendall trend tests, Continuous Wavelet Transform analysis, causal inference analysis) and qualitative (15 key stakeholder interviews) methods. The quantitative analysis employed monthly rainfall and temperature data (1991–2021) alongside RTC data (1998–2021) across 10 regions. While rainfall trends varied regionally, the wet season (April through mid-October) showed a strong link to crash severity for all regions across Ghana. Wavelet analysis showed higher crash severity in the wet season within every 2–8 months period in a particular annual year during the study period. Causal inference analysis revealed rainfall's stronger influence (3.59%) on fatal crashes during the wet season compared to temperature (0.04%). Key stakeholder interviews highlighted perceived changes in temperature and intense rainfall patterns affecting RTCs, especially during rainy seasons suggesting an association between increased rainfall and crash severity. These findings emphasize the multifaceted role of climate change on road safety and the need to address weather-specific risks.

## 1. Introduction

An estimated 1.19 million people died on the world's roads in 2021, a modest 5% decline from the 1.25 million fatalities recorded in 2010 globally (World Health Organization [WHO], 2023). This decrease occurred amidst a backdrop of concerning worldwide growth trends. However, Africa experiences a disproportionate burden of traffic fatalities. While the continent only has about 2% of the world's vehicles, it accounts for roughly one-quarter of all global traffic deaths (WHO, 2015). Though there are some positive outcomes from road safety initiatives, they remain insufficient to achieve the ambitious target outlined by the United Nations Decade of Action for Road Safety which aims to halve the number of Road Traffic Crash (RTC)-related deaths by 2030.

One significant factor influencing road safety is the road environment, which includes weather conditions (Eboli et al., 2020). Emerging evidence suggests a connection between extreme weather events and RTCs (Markolf et al., 2019; Braun & Fraser, 2022; Zare Sakhvidi et al., 2022). Studies have shown that high temperatures and heavy precipitation can increase the likelihood and severity of RTCs (Mansuri et al., 2015; Sadeghniiat-Haghighi et al., 2015; Shrestha et al., 2017). This intersection of road conditions with climatic factors emphasizes the broader impact of environmental changes on road safety. Climate change poses a major challenge to human and ecological systems globally (Parmesan et al., 2022). The Intergovernmental Panel on Climate Change (IPCC) predicts a rise in global mean temperatures of 1.4–5.8°C by 2100 (Kikstra et al., 2022). This warming trend is expected to disproportionately impact developing countries, particularly those in Africa (Kikstra et al., 2022).

Ghana is already experiencing the impacts of climate change through altered temperature patterns, variations in precipitation, and rising sea levels (Asante & Amuakwa-Mensah, 2014; Arndt et al., 2015;

Awuni et al., 2023). These changes threaten various socio-economic sectors, including transportation, agriculture and related livelihoods (Arndt et al., 2015; Chemura et al., 2020; Antwi-Agyei & Stringer, 2021; Baffour-Ata et al., 2021; Awuni et al., 2023). In Ghana, transportation is facilitated by various modes including road, rail, air, and water. Road transport is the primary carrier of both passengers and freight in Ghana, accounting for over 95% of traffic (Naazie et al., 2018). The road network includes trunk roads, urban roads, and feeder roads. Road transportation is crucial for Ghana's socioeconomic growth, facilitating the movement of people and goods (Asomani-Boateng et al., 2015). However, the country grapples with a high number of road traffic crashes (RTCs), with over 13,584 incidents recorded in 2020 alone, resulting in fatalities exceeding 2,080 and injuries surpassing 7,000 individuals (National Road Safety Authority [NRSA], 2020).

While traditional studies on RTCs in Ghana focus on factors like speeding and traffic violations Konlan et al. (2020), the potential influence of climate change on these crashes remains underexplored. This knowledge gap hinders the development of appropriate policies and interventions to address climate related RTCs in the country. By addressing this important research gap, this study aimed to examine the association between climate change and RTCs in Ghana. Specifically, the research questions guiding this paper are: (i) What is the trend of temperature and rainfall for the period 1991–2021 (ii)What is the relationship between rainfall, temperature and RTCs in Ghana? (iii) Does seasonality play a role in the occurrence of RTCs in Ghana? (iv) How do key stakeholders perceive the influence of climate change on RTCs?

### 2. Materials and methods

## 2.1 Description of study area

Ghana is in West Africa within the geographical coordinates 4°N and 11°N and 4°W and 2°E (Ministry of Food and Agriculture [MOFA], 2015). For greater clarity and context, it is important to note that Ghana increased its number of administrative regions from 10 to 16 in 2019. The data for this study was therefore collected prior to this administrative reorganization. At the time the data was gathered, Ghana was divided into ten regions and two hundred sixteen districts, encompassing five agroecological zones: Coastal Savannah, Evergreen, Deciduous Forest, Transitional, and Savannah (Online Resource 1) (Ghana Statistical Service [GSS], 2013). The country experiences a tropical climate influenced by its proximity to the equator, the Gulf of Guinea, and the West African Monsoon system (Yamba et al., 2023). This results in two main seasons: the wet season and the dry season.

The southern regions (Ashanti, Brong-Ahafo, Central, Eastern, Greater Accra, and Western) exhibit a bimodal pattern of rainfall distribution, while the northern regions (Northern, Upper East, and Upper West) have a unimodal pattern (Yamba et al., 2023). Seasonal changes impact road safety, making climate a critical factor to be considered for road safety management. Mean annual temperatures vary across the regions of Ghana, with the coastal areas having a different climate compared to the northern regions. The ecological diversity of Ghana plays a crucial role in understanding the impacts of climate change. The three northern regions, namely Upper West, Upper East, and Northern, are characterized by agroecological savannah landscapes, with a sparse population (GSS, 2013). In contrast, the Ashanti, Brong Ahafo, and Eastern regions feature transitional and deciduous forests, contributing significantly to the production of cocoa, lumber, and minerals. The Western area is dominated by evergreen and deciduous forests, hosting a bustling harbor, large- and small-scale mines, and offshore oil fields. A large portion of Ghanaians rely on rain-fed subsistence agriculture mostly transported by road. Given the significant variations in rainfall patterns and temperature levels across the country, understanding these variations at a local level is therefore crucial for road safety.

## 2.2 Data collection

## 2.2.1 Temperature and rainfall data

Daily rainfall, minimum and maximum temperature data were obtained from the Ghana Meteorological Agency for 30 years spanning the years 1991 to 2021 for 22 synoptic stations as shown in Online Resource 1. The period from 1991 to 2021 was selected to provide a comprehensive 30-year dataset for climate change analysis. The 22 synoptic stations were grouped and averaged to represent the corresponding region where the station is located. The analysis considered the previously established ten administrative regions as compared to the current sixteen for uniformity. This was performed to ensure consistency with road traffic crash data. Also, climate and crash data were aggregated to monthly temporal resolution.

## 2.2.2 Road traffic crashes data

This study utilized road crash data from 1998 to 2021 as this was the available data range, collected monthly by the Building and Road Research Institute (BRRI). The crash data is analyzed by coding and storing it in the Council for Scientific and Industrial Research [CSIR] – Building and Road Research Institute [BRRI] computers using the Micro-computer Accident Analysis Package software, which was designed by the Transport Research Laboratory (TRL) in the United Kingdom. The data captured goes beyond just RTC counts but includes other important details on crash severities: fatalities, injuries requiring hospitalization, minor injuries, and property damage. The "fatal crashes" involve at least one death within 30 days, "injured but not hospitalized" means receiving medical attention without an extended stay, and "serious injury" requires hospitalization for more than 24 hours.

## 2.2.3 Key stakeholder interviews

To validate the quantitative findings, a qualitative approach involving interviews with key road safety stakeholders was conducted to gather insights. Semi-structured interviews were used as a data collection method due to its ability to include a combination of predetermined questions and flexibility to accommodate individual differences among participants (Lune & Berg, 2017; Fylan, 2005). Purposive and convenience sampling were used to select the categories of the key stakeholders. Purposive sampling was chosen to guarantee a sufficient inclusion of the many individuals and groups that play a significant role in road safety and climate change relevant to the research questions and ensure diverse perspectives. They comprised four key stakeholder groups: governmental road safety and weather agencies, academics, road transport unions, and road law enforcement personnel. A total of 15 participants were interviewed, with the process reaching saturation after 11 interviews. The interviews, lasting 35–40 minutes each, employed a set of open-ended questions to obtain comprehensive responses. All interviews were audio-recorded with informed consent. To mitigate subjective bias, the study employed diverse and purposive sampling, semi-structured interviews, data saturation, audio recordings with informed consent, and independent data analysis.

### 2.3 Data analysis

### 2.3.1 Trend analysis of climate parameters (rainfall and temperature) and RTCs

The Mann-Kendall trend test as employed in Baffour-Ata et al. (2021) was utilized to assess trends in rainfall and temperature patterns across dry and wet seasons in Ghana. This non-parametric test evaluated climatic data for monotonic trends (increasing or decreasing) over time.

The Mann-Kendall trend test following Gilbert (1987) is calculated as follows:

$$S = \sum_{k=1}^{n-1} \sum_{j=k+1}^{n} sgn(x_j - x_k)$$

1

……………………

where $x_j$ *and* $x_k$ represent a sequence of values of variable understudy and *n* denotes the size of the time series. The $sgn(\theta)$ was evaluated from the difference between $x_j$ *and* $x_k$:

$$sgn(\theta) = \begin{cases} 1 & if\,\theta > 0 \\ 0 & if\,\theta = 0 \\ -1 & if\,\theta < 0 \end{cases}$$

2

……………………..

Coefficient of Variation (CV) is mathematically expressed as follows:

$$CV = \frac{\sigma}{\mu}$$

3

……………………………….

where the $\sigma$ and $\mu$ are the standard deviation and mean of the climate variables and seasonal RTCs. Higher values of CV often denote high variation in the observed variable which makes variable predictability difficult. The analysis was performed in Python using the pymannkendall packages.

### 2.3.2 Continuous wavelet transform

Continuous wavelet transform analysis, a non-stationary method, was used to analyze the time-frequency localization of RTC data. This method, unlike time series analysis, provides insights into both time and frequency, revealing when and at what frequencies specific events occur. The Morlet mother wavelet, known for its time-frequency localization properties, was chosen for this analysis.

$$\psi_0(\eta) = \pi^{\frac{-1}{4}} e^{i\omega_0\eta} e^{\frac{-\eta^2}{2}}$$

4

……………………….

Where $\psi_0(\eta)$ denotes estimated wave value at the non-dimensional time $(\eta)$ while $\omega_0$ represents the mother wavelet's non-dimensional frequency. This basic wavelet function creates the fundamental step to develop a scaled wavelet that allows the entire wavelet to slide along time as shown in Eq. 5:

$$\psi\left[\frac{(n' - n)\delta t}{s}\right] = \left(\frac{\delta t}{s}\right)^{\frac{1}{2}} \psi_0\left[\frac{(n' - n)\delta t}{s}\right]$$

5

………………………

Therefore, for a discrete sequence $x_n$ which may represent the road traffic severities, a continuous wavelet transform is defined as the convolution of $x_n$ with a scaled and a translated version of $\psi_0(\eta)$:

$$W_n(s) = \sum{}_{n'}^{N-1} x_{n'} \psi^*\left[\frac{(n' - n)\delta t}{s}\right]$$

6

……………………

where * is a complex conjugate. By varying the wavelet scale (s) and sliding along the localized time index $(n)$ for the various RTC severities, one can create an image that presents the amplitude of any characteristics identified against how these characteristics change with time.

### 2.3.3 Causal relationship between climate variables and road traffic crashes

Causal inference analysis, a method that goes beyond correlation statistics, was employed to investigate the causal influence of climate variables (temperature and rainfall) on RTCs. The Do-Why Graphical Causal Model software was used to construct a causal model graph depicting the assumed relationships between these variables. Functional causal models (FCMs) were established for non-root nodes, while stochastic models were used for root nodes (rainfall and temperature) to define causal mechanisms.

**H - Hospitalized; I - Injured; D - Damage; F – Fatal; TRTC – Total Road Traffic Crashes**

In addition to the causal graph (**Online Resource 2)**, a causal mechanism is generated for each of the variables with a general form;

$$Y = f\left(PA\right) + \epsilon$$

7
…………………….

where $PA$ refers to the variable's causal parent node and $\epsilon$ is the noise independent of the observed parent. Here $f$ is a potential nonlinear or linear function that determines the value of $Y$ based on the causal parent's values. The causal mechanisms were defined by functional causal models (FCMs) for non-root nodes and stochastic models for root nodes. The two root nodes (Rainfall, Temperature) are defined as:

$$Rain\ := Rain_x$$

8
………………………..

$$Temp\ := Temp_x$$

9
……………………..

Therefore, the structural equation that governs the structural causal model for which inferences are drawn is presented as follows:

$$H \coloneqq f\left(Rain,\ Temp\right) + n_H$$

10
………………

$$I \coloneqq f\left(Rain,\ Temp\right) + n_I$$

11
………………..

$$D \coloneqq f\left(Rain,\ Temp\right) + n_D$$

12

…………………

$$F \coloneqq f\left(Rain,\ Temp,\ H, I\right) + n_F$$

13

………….

$$TRTC \coloneqq f\left(H, I, D, F\right) + n_{TRTC}$$

14

………….

Based on the probability causal model developed, information of how strong a causal influence is from a cause to its direct effect is determined.

#### 2.3.4 Thematic analysis of qualitative data

The key stakeholder interviews were audio-taped and transcription was conducted and reviewed to guarantee accuracy. The online transcription program "Otter.ai" was used to auto-generate the initial transcripts, which were then downloaded and cleaned in Microsoft Word Version 23 to match the recording exactly. Thematic analysis was used to identify the common themes and patterns in the stakeholders' responses. Thematic analysis is a methodological approach used to analyze qualitative data, in which the researcher systematically examines the data to uncover recurring themes (Castleberry & Nolen, 2018). The transcribed interviews were reviewed, and relevant excerpts were selected to highlight key points from the interviews.

### 3. Results

#### 3.1 Trends and variabilities in rainfall and temperature

The analysis of rainfall patterns revealed a potential shift in climatic conditions across Ghana. Online Resource 3 depicts monthly rainfall trends observed during the wet season (1991–2021). While all regions received between 0 and 300 mm of monthly rainfall, the Upper East Region consistently recorded the lowest amounts (0–80 mm). Rainfall exhibited decreasing trends during the wet season in eight out of the ten regions. However, these trends were not statistically significant ($p > 0.05$). Conversely, most regions showed increasing trends in monthly rainfall during the dry season, although these trends were also statistically insignificant ($p > 0.05$). Notably, all regions exhibited high coefficients of variation (CV > 50%) for rainfall, indicating unpredictable and erratic rainfall patterns (Online Resource 3 and 4).

In contrast to rainfall patterns, surface temperature across all ten regions showed statistically significant increases ($p < 0.05$) during both wet and dry seasons (Online Resource 5 and 6). The rate of temperature increase ranged between 0.0001°C/month and 0.0045°C/month during the wet season, with the Northern Region experiencing the most significant rise. The dry season witnessed a more pronounced increase in temperature, with the Brong Ahafo Region exhibiting the highest rate of change (0.029°C/month). This finding is also consistent with some emerging themes from the responses from some key stakeholders who noted that the temperature trends in Ghana have increased and that rainfall patterns have become more erratic and intense over the past three decades. They reported changes in temperature, and variations in rainfall onset, intensity, and amount. A key stakeholder noted that:

*"[...] Yes, from the available data, there have been significant changes in these parameters. There have been extreme cases of rainfall where there have been short but heavy downpours. There are variations with the seasons with August being coldest, March being hottest and January having the peak of the harmattan even though harmattan has not been drastic in recent years"*- (Key stakeholder, September 2023)

### 3.2 Spatial distribution and severity of road crashes in Ghana

The results of the road traffic crash (RTC) analysis revealed a distinct spatial distribution pattern. Online Resource 7

is the mean annual total RTCs recorded by the Building and Road Research Institute (BRRI) from 1998 to 2021. During this period, Ghana experienced an average of 10,556 RTCs annually, with a higher concentration observed in the southern regions compared to the north. Greater Accra (4,423 crashes), Ashanti (1,832 crashes), and Eastern Region (1,241 crashes) emerged as the top three contributors to national RTCs. Conversely, the Upper West (102 crashes), Upper East (147 crashes), and Northern Region (230 crashes) recorded the fewest incidents (Fig. 1).

Figure 2 shows the percentage distribution of RTC severity (fatal, injury requiring hospitalization, injury not requiring hospitalization, and property damage only) across the ten regions. The findings highlight significant regional variations in crash severities. For instance, Greater Accra, despite recording the highest number of crashes, recorded a relatively low fatality rate (8%, $n$ = 354 crashes annually). Property damage, on the other hand, constitutes the most frequent outcome in this region, accounting for approximately 50% ($n$ = 2,212 crashes) of all incidents. Injuries requiring hospitalization and those not requiring hospitalization occur at moderate frequencies of 19% and 25%, respectively.

The distribution patterns in Ashanti and Eastern Regions deviate from that of Greater Accra. In the Ashanti Region, approximately 30% ($n$ = 550 crashes) of incidents result in hospitalized injuries, while fatalities account for 18% ($n$ = 330 crashes) annually. Injuries not requiring hospitalization and property damage hover around 24% and 25% of the average annual RTCs in this region. The Eastern Region, the third-highest contributor to RTCs, displays another distinct pattern. While fatalities remain relatively low at 18% annually, hospitalized injuries take precedence, constituting 32% of the crashes recorded during the study period. These findings emphasize the importance of considering regional variations in both the frequency and severity of RTCs when developing targeted road safety interventions.

### 3.3 Seasonality and variability of road crash severity in Ghana

The result of the seasonal patterns and variations in road traffic crash (RTC) analysis revealed a consistent trend across most regions: higher variations in total RTCs and their severity occur on a periodic scale of 2–8 months (Fig. 3, Online Resources 7 and 8). These recurring patterns suggest seasonal dynamics in both the overall number of RTCs and the distribution of crash severity (fatal, hospitalized injuries, injuries not requiring hospitalization, and property damage only).

Regions with the highest RTCs (Greater Accra and Ashanti) exhibited similar patterns in the variation along the 2–8-month band. Notably, for Greater Accra, these significant variations (indicated by black contours) concentrated in the early years (2000–2003) across all crash severity categories (fatal, injury, property damage, and hospitalized victims). In contrast, regions like Brong Ahafo and Central displayed more prominent variations throughout the study period (Fig. 3). The Volta, Eastern, Northern, Upper East, and Upper West Regions exhibited stronger wavelet power variance compared to the Ashanti and Eastern Regions. The 2–8-month band contained several significant variations from 1998 to 2021. A key stakeholder spoke frankly about the realities of driving in such seasons:

*"During dry seasons we experience foggy conditions which affect visibility. During rainy seasons, especially the short frequent ones, the roads are submerged destroying the road condition and causing erosion leading to the development of potholes and loss of control in vehicles. For example, on the Tema Motorway, anytime it rains the roads become slippery and we normally record a high number of vehicles losing control and skidding off the road. Also, the Mallam-Kasoa Highway, anytime it rains heavily, mudslides are experienced on that road"* (Key stakeholder, October 2023)

### 3.4 The relationship between climate variables and road traffic crashes severity in Ghana

Rainfall showed a causal strength (3.59%) on accidents that were fatal while it showed a causal strength (46.72%) when considering damages from road crashes (Fig. 4). Rainfall showed a higher causal strength (1.44%) towards fatal road crashes compared to temperature (0.28%) during the dry season in Ghana (Fig. 5). Temperature during the dry season showed strong causal strength (71.54%) towards damage severity. This result suggests that climate variables truly impact road traffic crashes in Ghana. The result showed a causal mechanism between the climate variables on road crash severity. This highlights that the impact of the climate parameters (temperature and rainfall) differs in various regions. It is observed that for different severities (damage only, injury, hospitalized and fatal), rainfall showed different causal effect contributions from the variance in the data. For instance, rainfall expressed a stronger causal effect on road accidents that led to damages in all the regions but showed different contribution factors as compared to temperature. General observations showed that during the wet season, the majority of the road crashes that damaged (D) properties may have rainfall as one of the factors. These same observations can be seen in the Greater Accra, Central, Northern, Upper East and

West Regions. Rainfall also remains one of the factors that influenced accident that hospitalized victims in four regions (Central, Western, Northern and Volta Regions). Similar to the damage only severity during the wet season, rainfall predicted higher causal strength towards crashes that were fatal in several regions during the wet season (see Table 1).

In the dry season, very few regions presented rainfall as one of the factors that influenced or caused accidents that damaged (Central, Western, Northern and Upper West Regions) properties as well as fatal incidents (Greater Accra, Ashanti, Central and Upper West Regions). From the causal inference model, the results suggest that temperature has strong causal strength on the various accident crashes that occurred in the dry season each year.

Again, the model points out that rainfall resulted in more fatal road crashes in all the regions except Greater Accra, Eastern and Upper West Regions. Although temperature presented a stronger causal effect on accidents that resulted in injuries when compared to rainfall, it is important to highlight that rainfall shows indirect causal effects through fatal accidents on injuries sustained during accidents in all regions.

It is apparent that Greater Accra, the most densely populated region in Ghana, exhibited a particularly strong association between the climatic factors and road crash severity. In this region, the causal contribution of rainfall and temperature to damage, injury, hospitalization, and fatality were 63.93;36.06, 2.4;11.24, 0.91;0.14, and 40.95;59.03, respectively. On the other hand, the Ashanti and Central Regions also displayed substantial causal relationships between climate variables and road crash severity across various categories. A key stakeholder expressed the same finding:

*"The number one enemy of the road is water, and the flooding of our roads brought on by intense and frequent rainfall has increased due in part to poor construction of drainage systems and culverts. This poses a road safety hazard as water ponds on the road and cause potholes. For example, the Duampopo road on the Accra-Kumasi Road, the New Koforidua Road and the Adansi Road. Also, constant rainfall leads to the faster growth of grasses around roads impeding vision on the shoulders of the road further narrowing the road. During rainfall, the roads also become slippery, resistance is low and even the application of the brakes can fail leading to the skidding of cars off roads which impacts the severity of the crashes"* - (Key stakeholder, October 2023)

## 4. Discussion

## 4.1 Rainfall and temperature trends in Ghana

Results revealed significant shifts in rainfall patterns towards drier periods with increased variability and unpredictability during the wet season in certain regions, aligning with stakeholders' observations of diminishing distinct seasonal boundaries compared to previous years. This trend is consistent with prior research by (Abbass et al., 2022; Antwi-Agyei et al., 2021; Atiah et al., 2021), illustrating Ghana's progressing aridity and susceptibility to drought conditions over the past century. High rainfall variability, characterized by a coefficient of variation (CV) exceeding 50% across all regions, poses challenges for road infrastructure upkeep and management, contributing to hazardous driving conditions like potholes, mudslides, and flash floods, which elevates crash risks. Temperature analyses reveal regional warming trends, notably in Northern Regions where temperatures are rising significantly (0.029°C/month), potentially impacting road traffic crash occurrences. These findings underline the increasing temperatures across Ghana during both wet and dry seasons, with rainfall changes exhibiting greater variability, aligning with forecasts by the Environmental Protection Agency, Ghana [EPA] (EPA, 2021) predicting continued temperature rises alongside shifting rainfall patterns.

## 4.2 Trend and seasonality (dry and wet seasons) of road traffic crashes in Ghana

Findings highlighted distinct seasonal patterns in road crash severity, notably higher during the wet season compared to the dry season, aligning with stakeholders' observations of increased road crashes during rainy periods, particularly in the Ashanti and Upper East Regions. These trends are consistent with (Liu et al., 2017), who noted that rainfall can elevate traffic accident rates by affecting road surface traction and vehicle control. Wet conditions exacerbate risks, especially on poorly constructed roads with inadequate drainage, leading to skidding and increased crash severity. Moreover, time-frequency analysis revealed a consistent periodicity (2–8 months) in road crash severity across all regions, suggesting recurring patterns possibly linked to rainy seasons and festive periods. Stakeholder insights further emphasized these seasonal dynamics, attributing them to factors like substandard road construction, irregular maintenance, inadequate drainage, urban expansion, and intense rainfall events within short durations. These multifaceted factors contribute to the observed seasonality and variability in road crash severity, highlighting the complex interplay between weather events, road conditions, and RTCs.

## 4.3 The relationship between road traffic crashes, rainfall and temperature in Ghana

Findings reveal varying impacts of climate parameters, specifically temperature and rainfall, on RTCs across different regions of Ghana. Notably, rainfall exhibits a causal influence (3.59%) on fatal crashes during the wet season compared to temperature (0.04%), contributing to slippery road conditions and reduced visibility as confirmed by the key stakeholders. The Greater Accra region notably demonstrates a robust association between climatic factors and road crash severity, particularly due to flash floods resulting from intense rainfall events (Gaisie & Cobbinah, 2023; Osei et al.,2023). Similarly, the Ashanti and Central regions show significant causal relationships between climate variables and RTC severity, whereas the Upper East and Upper West Regions exhibit weaker associations, possibly attributed to regional climate variations and socio-economic factors. This is consistent with studies by (Yun Yuan et al., 2014; Sun & Dong, 2022). Understanding these climate-related risks provides the need for targeted interventions and road safety measures tailored to regional climatic conditions to reduce the incidence and severity of RTCs associated with adverse weather events.

## 5. Conclusion

This study examined the impact of climate change on RTCs in Ghana. It employed a mixed-method approach, combining rainfall, temperature and RTC data with interviews from key stakeholders. The findings confirm that key stakeholders perceived changes in climate, with more erratic rainfall patterns and rising temperatures which may influence the occurrences of RTCs. These observations align with the analyzed climate data. The study revealed that variations in temperature and rainfall can have differing impacts on both the frequency and severity (injuries, fatalities, damages) of RTCs. There is a strong seasonal trend, with a period of heightened risk lasting 2–8 months per year. The regions most affected are Greater Accra, Ashanti, and Eastern. Importantly, the impact of climate on RTCs varies across regions. Some regions, like Greater Accra, show a strong link between weather and crash severity. In contrast, regions like the Upper East and Upper West show weaker connections. This highlights the need for region-specific road safety strategies. It is also revealed that rainfall has a stronger influence on fatal crashes during the wet season compared to temperature. This suggests a positive correlation between increased rainfall and crash severity. The study acknowledges the multifaceted role of climate on road safety. Recognizing the varying levels of negative influence climate variables have on RTCs can inform practical planning for more sustainable solutions to improve road safety and adapt to a changing climate.

Based on these findings, the following recommendations are proposed. Regions should leverage data-driven insights to develop targeted interventions and risk maps. This data can inform strategies for managing crashes in high-risk areas, including updating road construction standards, and incorporating rainfall and temperature forecasts into road planning and policy tools. Further, the National Road Safety Authority (NRSA), the Building and Road Research Institute (BRRI), and other key stakeholders can collaborate to establish driver education programs that address safe driving practices in various weather conditions, such as rain, fog, and heat. Integrating such education into community plans or other relevant strategies can encourage drivers to adjust their behavior and speed in response to weather-related hazards like reduced visibility. Finally, stakeholder feedback highlighted the limitations faced by local governments due to inadequate resources and policy constraints in implementing coping and adaptation strategies to reduce crashes, particularly during rainy seasons. Increased funding, guidance, and human resources would significantly improve their ability to carry out these crucial functions. By implementing these recommendations and acknowledging the regional variations in climate change impact on RTCs, Ghana can move towards a more sustainable and resilient approach to the impacts of climate change on road safety.

## 6. Future research directions

It is suggested that future research explores the impact of climate change on RTCs using different sets of climatic data such as humidity and windspeed data. Also, areas such as rainfall only on paved and unpaved road areas or only heavy-frequency rainfall events case studies, as well as data with more control variables, such as demographic, cultural, and socioeconomic features could be employed.

#### Limitations of the study

As data are extracted from a compilation of police files using a standard crash reporting form which is then coded and stored in computers at the CSIR-BRRI, which is one of the best established RTC databases in sub-Saharan Africa, data quality can be assured. However, it is important to note that the crash database is subject to some level of underreporting which includes both non-reporting and under-recording. Under-recording represents the shortfall in recovery (under-recovery) of data on the number of crashes from police files. Non-reporting, on the other hand, is when the police are not notified at all of the occurrence of a road crash. Second, in the current study, traffic violation types were not separated for additional analysis. Speeding, intoxicated driving, and tired driving are all substantial risk factors. Despite these limitations, the results of this paper's analysis of the long-term cumulative changes in climate and its impact on RTCs support the existing body of evidence that shows the linkage between climatic variables and RTCs severity.

### Declarations

Acknowledgements

The authors express their gratitude to the Ghana Meteorological Agency (GMet) and the Building and Road Research Institute (BRRI) of the Council for Scientific and Industrial Research (CSIR), Ghana for generously providing the relevant data, and extend their appreciation to the key stakeholders that were interviewed.

#### Funding

This study was supported by grant D43 TW007267 from the Fogarty International Center at the US National Institutes of Health. The content is solely the responsibility of the authors and does not necessarily represent the official views of the NIH.

#### Ethical considerations

Ethical clearance and approval were obtained from the Kwame Nkrumah University of Science and Technology (KNUST) Humanities and Social Sciences Research Ethics Committee (HuSSREC/AP/144/VOL.2).

#### Declaration of competing interest

The authors declare that they have no known competing financial interests or personal relationships that could have appeared to influence the work reported in this paper.

#### Data availability

The datasets generated during and/or analyzed during the current study are available from the corresponding author on reasonable request.

## Table 1

**Table I Summary of the causal contribution (%) of rainfall and temperature to road crash severity in the 10 regions during the wet and dry season**

| Region | Road Crash Severity | | | | | | | |
|---|---|---|---|---|---|---|---|---|
| | Wet season | | | | Dry Season | | | |
| | D (Rain; Temp) | I (Rain; Temp) | H (Rain; Temp) | F (Rain; Temp) | D (Rain; Temp) | I (Rain; Temp) | H (Rain; Temp) | F (Rain; Temp) |
| Greater Accra | 71.72; 28.27 | 36.77; 63.22 | 10.05; 89.94 | 3.20; 0.49 | 32.67; 67.32 | 8.62; 91.37 | 7.43; 92.56 | 6.22; 2.26 |
| Ashanti | 5.02; 94.97 | 2.70; 97.29 | 3.85; 96.14 | 0.70; 0.24 | 21.57; 78.42 | 98.52; 1.47 | 31;96; 68.03 | 0.62; 2.40 |
| Central | 60.97; 39.02 | 81.80; 18.19 | 89.86; 10.03 | 4.62; 1.70 | 65.97; 34.02 | 16.30;83.69 | 60.61; 39.38 | 21.35; 0.18 |
| Eastern | 43.47; 56.52 | 27.53; 72.46 | 46.99; 53.00 | 10.94; 2.37 | 28.51; 71.48 | 5.66; 94.33 | 15.6; 84.39 | 4.83; 4.98 |
| Western | 33.05; 66.94 | 27.53; 72.46 | 53.57; 46.42 | 1.78; 7.80 | 68.10; 31.89 | 67.37; 32.62 | 61.04; 38.95 | 4.79; 0.54 |
| Brong Ahafo | 5.21; 94.78 | 19.49; 80.50 | 38.85; 61.14 | 2.96; 3.52 | 12.75; 87.24 | 11.88; 88.11 | 25.27; 74.72 | 3.35; 8.14 |
| Northern | 82.88; 17.11 | 78.07; 21.92 | 82.81; 17.18 | 7.42; 26.15 | 52.27; 47.72 | 28.97; 71.02 | 86.34; 13.65 | 1.29; 9.91 |
| Upper East | 65.34; 34.65 | 75.61; 24.38 | 15.31; 84.68 | 33.32; 47.47 | 8.15; 91.84 | 88.51; 11.48 | 35.76; 64.23 | 1.06; 66.13 |
| Upper West | 53.01; 46.98 | 39.45; 60.54 | 12.45; 87.54 | 11.52; 6.19 | 73.29; 26.29 | 16.31; 83.68 | 12.18; 87.81 | 43.43; 20.84 |
| Volta | 80.65; 19.34 | 17.53; 82.46 | 72.96; 27.03 | 1.51; 5.07 | 20.57; 79.42 | 25.70; 74.29 | 39.76; 60.23 | 1.08; 14.68 |

**D-Damage; I-Injury; H- Hospitalised; F-fatal**

## Figures

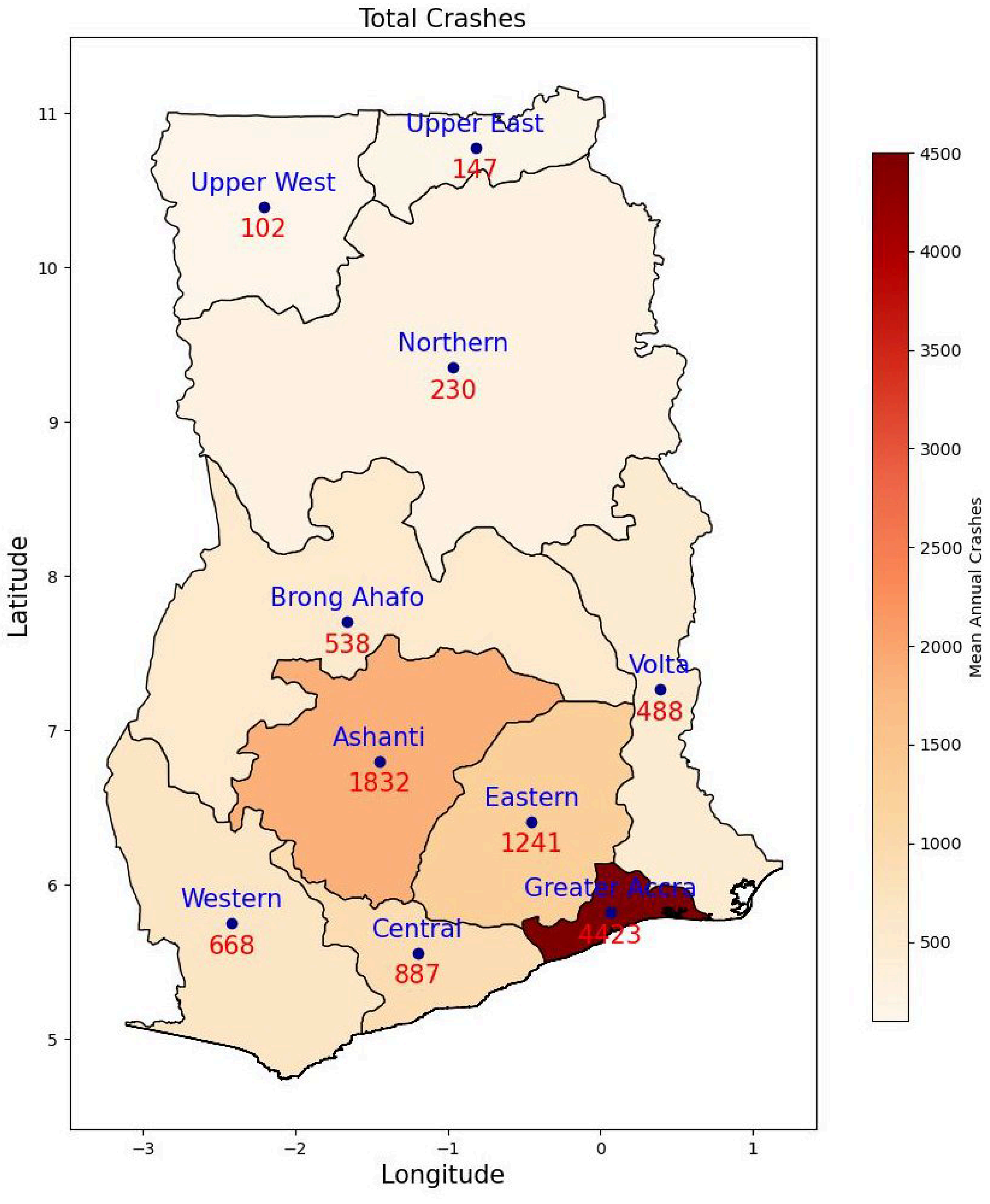

Figure 1

Mean annual regional distribution of RTCs in Ghana from 1998 to 2021

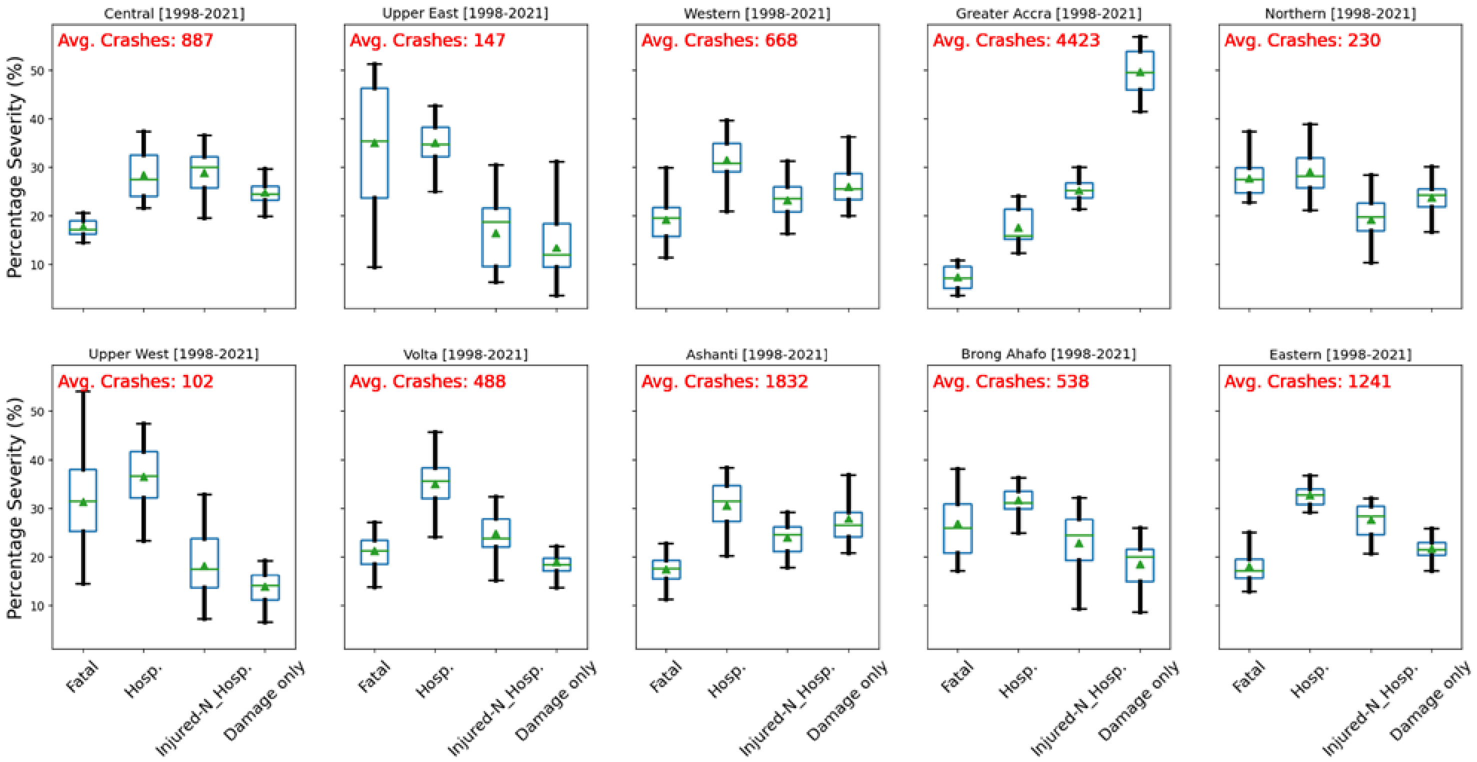

### Figure 2

### Percentage distribution of various severity of annual road crashes over Ghana

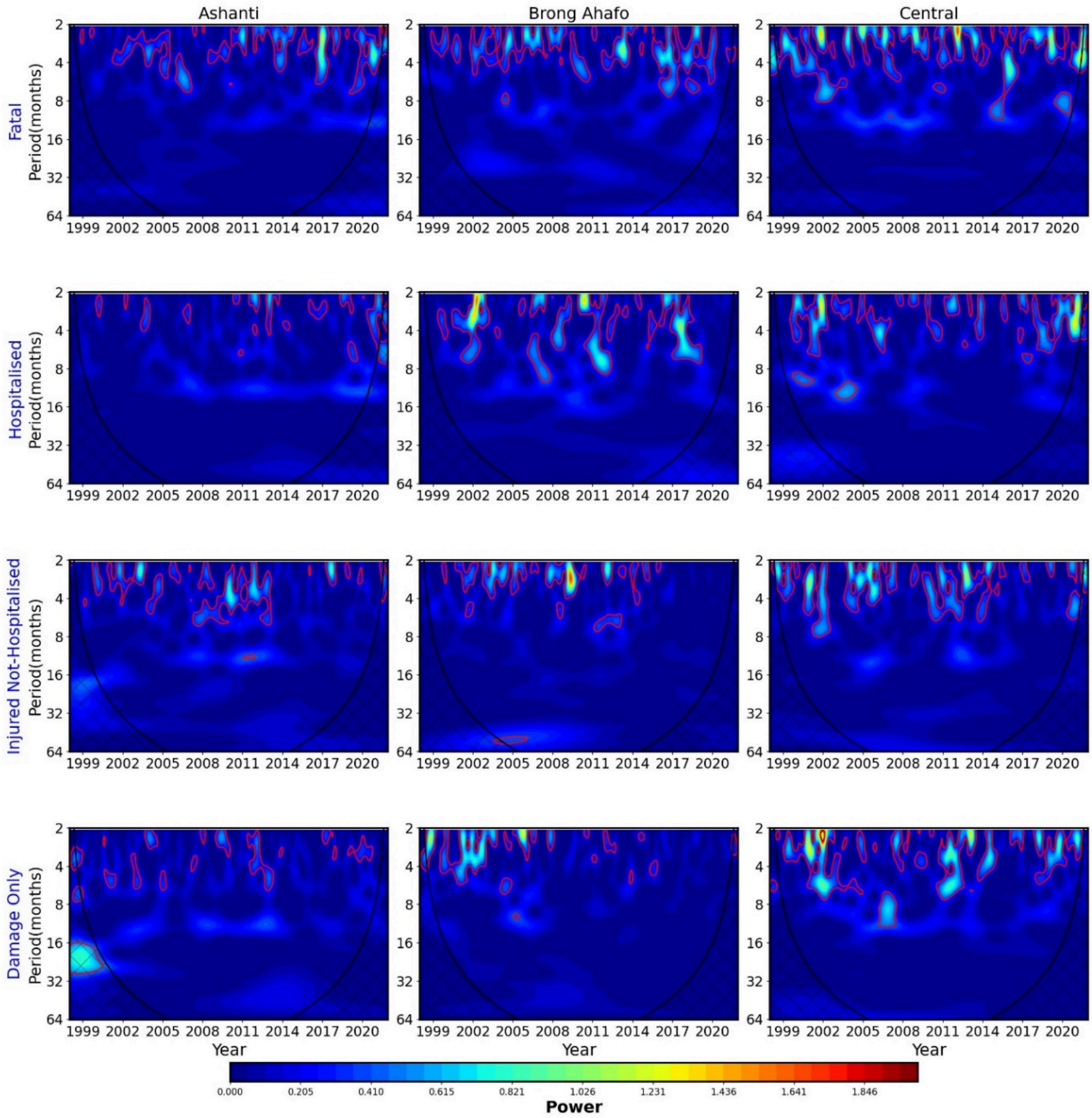

### Figure 3

### Wavelet power spectrum of total road crash and severity (row) for the various regions in Ghana (column)

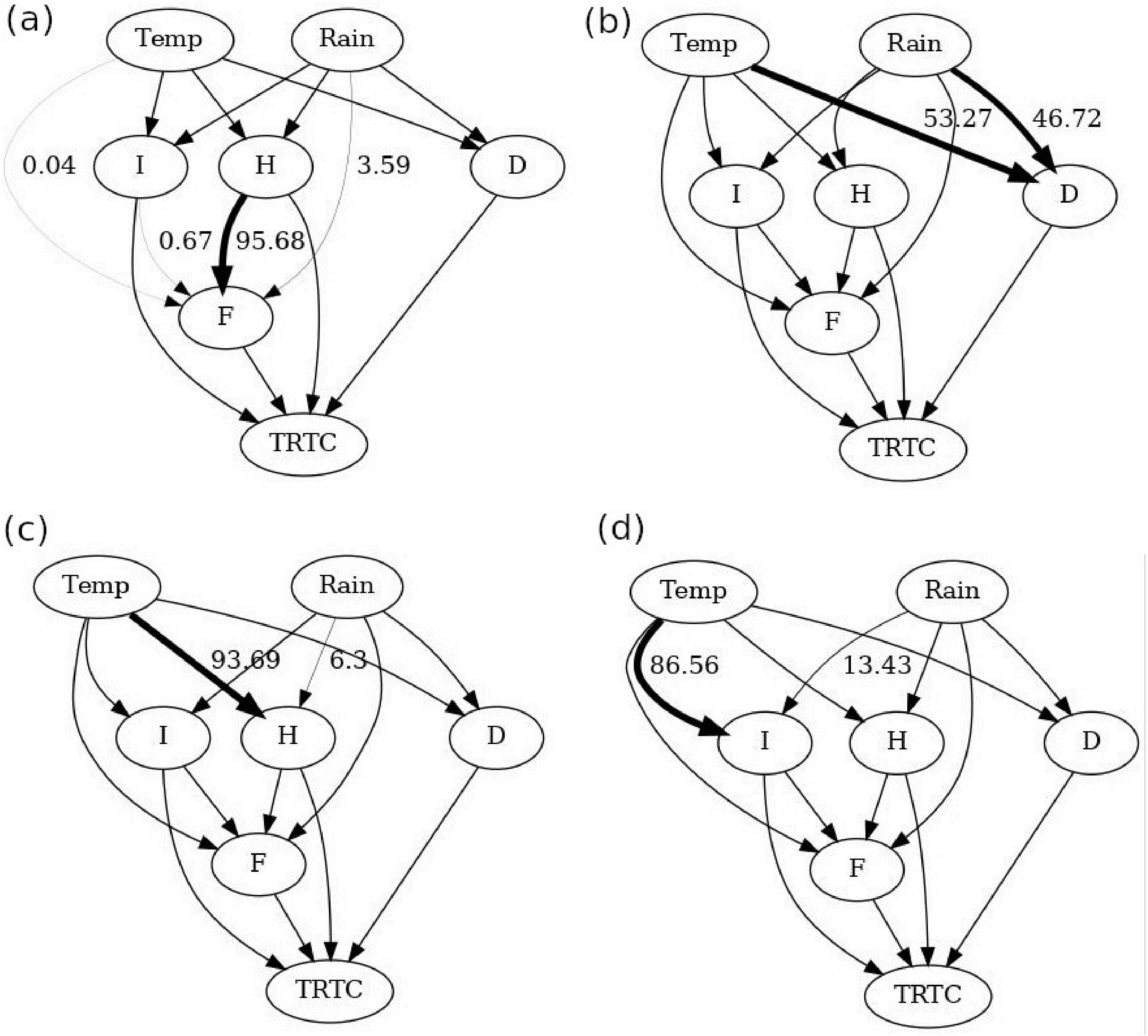

Figure 4

Percentage causal strength contribution of rainfall and temperature to Fatal (a), Damages (b), Hospitalised (c) and Injured (d) road crash severity during the wet season

H - Hospitalized; I - Injured; D - Damage; F – Fatal; TRTC – Total Road Traffic Crashes

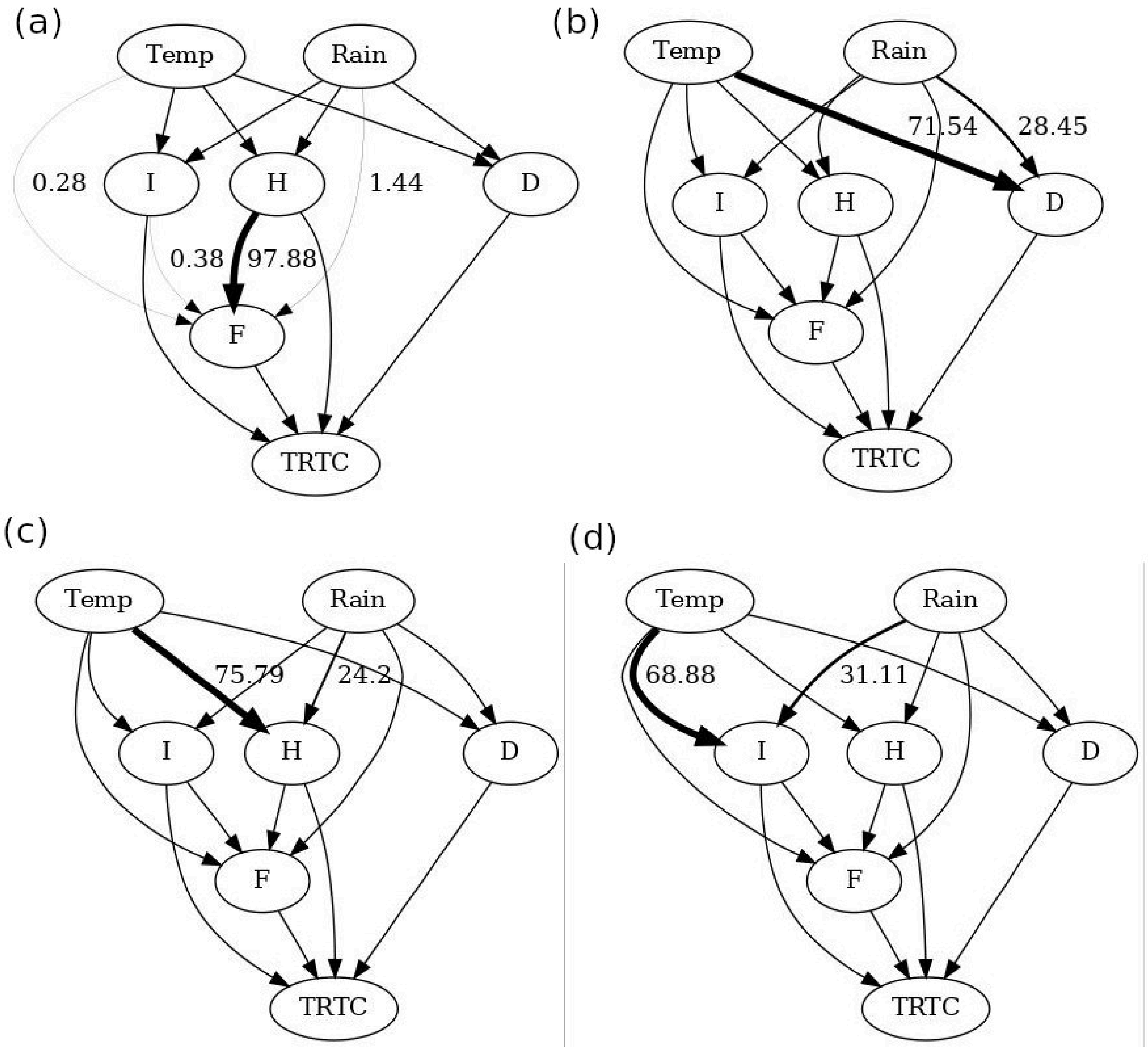

**Figure 5**

**Percentage causal strength contribution of rainfall and temperature to Fatal (a), Damages (b), Hospitalised (c) Injured (d) road crash severity during the dry season**

**H - Hospitalized; I - Injured; D - Damage; F – Fatal; TRTC – Total Road Traffic Crashes**

## Supplementary Files

This is a list of supplementary files associated with this preprint. Click to download.

- ESM1.pdf
